# Editorial: Insights into the relationships between host and vector microbiota, host health and response to disease

**DOI:** 10.3389/fvets.2022.1002247

**Published:** 2022-08-16

**Authors:** Dasiel Obregon, Alejandra Wu-Chuang, Alejandro Cabezas-Cruz

**Affiliations:** ^1^School of Environmental Sciences, University of Guelph, Guelph, ON, Canada; ^2^ANSES, INRAE, UMR BIPAR, Laboratoire de Santé Animale, Ecole Nationale Vétérinaire d'Alfort, Maisons-Alfort, France

**Keywords:** microbiota, ticks, infectious diseases, persistent pathogen infection, *Escherichia coli*

Microbes are often nested in large populations and have the capacity for rapid and continuous evolutionary change, which allows them to quickly adapt to diverse environments. They have evolved to live in mutualism with numerous animal species from arthropods (1) to mammals (2). Recent research has expanded our understanding of how bacterial communities within animals, regarded as microbiota, facilitate host protection (2) or disease (3). When invading a host, pathogens interact with other microbial species and modulate the microbiota (2, 3). Abiotic and biotic environmental factors (including the microbiota) influence disease dynamics (4), yet the importance of the host-microbiota-pathogen interactions has been poorly characterized in clinical settings.

## Microbiota modulation in clinical disease, persistent pathogen infection, and postpartum udder skin

In this Research Topic, Gomez et al. showed that diarrhea, a leading cause of morbidity, and mortality in calves; alter the bacterial communities of the gastrointestinal tract. The main relevance of the study lies in the elucidation of microbiota changes associated with anion gap acidosis (due to increased concentrations of anions including D- and L-lactate), a metabolic disorder frequently associated with acidosis caused by diarrhea. In particular, the authors found a shift from obligate anaerobes to facultative anaerobes in diarrheic calves. Facultative anaerobes such as bacteria of the family Enterobacteriaceae induce inflammatory response in animal models (5). Gastrointestinal inflammation increases the release of oxygen-rich haemoglobin within the gut lumen, which in turn favours colonization of facultative anaerobes (6). This positive feedback loop may contribute to the proliferation of lactate-producing bacteria including *Lactobacillus, Streptococcus, Veillonella, Ligilactobacillus* and *Olsenella* also enriched in diarrheic calves (4). By pointing at specific microbiota alterations potentially underlying the systemic acidosis and inflammation observed in diarrheic calves, the study by Gomez et al. opens the possibility of controlling clinical disease by preventing bacterial dysbiosis.

On the other hand, the longitudinal study presented by Bibbal et al. assessed the persistence of enterohemorrhagic *Escherichia coli* (EHEC) O157:H7 in 13 cattle farms; specifically, in one of the farms, they compared fecal microbiota between shedders (S) and non-shedders (NS) young bulls. The study found no significant differences in diversity and composition of the fecal microbiota between S and NS, but interestingly identified six amplicon sequence variants (ASVs), mostly belonging to Firmicutes phylum, which were indicators of shedders status, whereas another four ASVs were indicators of the NS group, which were affiliated *Pirellulaceae-1088-a5* gut group, *Anaerovibrio, Victivallis*, and *Sellimonas* genera. These results reveal the gut microbiota bioprospection as a promising research field for the discovery of next-generation direct-fed microbials (DFMs), which should help prevent the colonization of *E. coli* O157:H7, but also have beneficial immunomodulatory effects and contribute to animal nutrition (7).

The importance of host microbiota on the health-disease process was also reinforced by the study by Zhang et al., who analyze the udder skin microbiota of yak and cattle during the perinatal period. These authors showed that the microbial richness of bovine udder skin during 1–2 weeks postpartum was significantly lower than those in the 1–2 weeks prenatal and 1-month postpartum period; particularly the effects of the perinatal period on the udder skin microbial community were more significant in cattle compared to yak. Despite preliminary, this publication adds new pieces of evidence of the disturbing effects of peripartum on udder skin microbiota, which should be tested in association with the presence of pathogens causative of mastitis. The understanding of the association between udder skin microbiota disturbance and the susceptibility to mastitis can help in the prevention of this important disease, for instance, several studies have reported that udder commensal microbiota can modulate the susceptibility to intramammary infection by mastitis pathogens (8, 9).

## The tempo of bacterial microbiota in arthropod vectors

Animal and human pathogens that are transmitted by arthropods are a global concern, particularly those vectored by ticks (e.g., *Borrelia burgdorferi* and tick-borne encephalitis virus). Several factors shape the composition of the bacterial microbiota in ticks and they include abiotic (e.g., temperature) and biotic factors (e.g., tick species, host blood-meal, and tick-developmental stages) (1). With few exceptions (10, 11), studies of tick microbiota focus on bacterial community composition in single-time points, which limits the understanding of potential fluctuations across time. In their study, Perveen et al. followed the microbiota dynamics in the tick *Hyalomma dromedarii* for a year. Temporal fluctuations of microbial community composition and variability in the abundance of different bacterial groups were revealed. Interestingly, some bacteria genera such as *Staphylococcus, Bacillus, Francisella*, and *Corynebacterium* were present in all months, while *Brachybacterium* was replaced by *Acinetobacter* in the winter months. The results suggest that microbial populations within ticks may be more dynamic than previously thought. However, the persistence of some genera across time suggests transovarial transmission of the microbiota or conserved and persistent patterns of microbe acquisition. Considering that microbiota influence arthropod fitness and vector competence (1), ubiquitous bacteria in the *H. dromedarii* microbiota could be considered for their inclusion in anti-microbiota vaccines (12) to block the transmission of pathogens such as *Theileria* spp., *Rickettsia* spp., *Francisella* spp., and Crimean-Congo hemorrhagic fever virus transmitted by this tick species to animals and humans.

## Author contributions

All authors listed have made a substantial, direct, and intellectual contribution to the work and approved it for publication.

## Funding

UMR BIPAR was supported by the French Government's Investissement d'Avenir program, Laboratoire d'Excellence Integrative Biology of Emerging Infectious Diseases (Grant No. ANR-10-LABX-62-IBEID). AW-C was supported by Programa Nacional de Becas de Postgrado en el Exterior Don Carlos Antonio López (Grant No. 205/2018).

## Conflict of interest

The authors declare that the research was conducted in the absence of any commercial or financial relationships that could be construed as a potential conflict of interest.

## Publisher's note

All claims expressed in this article are solely those of the authors and do not necessarily represent those of their affiliated organizations, or those of the publisher, the editors and the reviewers. Any product that may be evaluated in this article, or claim that may be made by its manufacturer, is not guaranteed or endorsed by the publisher.
